# Development and evaluation of a standardized peer-training in the context of peer review for quality assurance in work capacity evaluation

**DOI:** 10.1186/s12909-018-1233-z

**Published:** 2018-06-13

**Authors:** André Strahl, Christian Gerlich, Georg W. Alpers, Katja Ehrmann, Jörg Gehrke, Annette Müller-Garnn, Heiner Vogel

**Affiliations:** 1 Department of Medical Psychology, Medical Sociology, and Rehabilitation Sciences, University of Würzburg, Klinikstraße 3, 97070 Würzburg, Germany; 2 Department of Orthopaedics, University Medical Center Hamburg-Eppendorf, Martinistraße 52, 20246 Hamburg, Germany; 3 Department of Psychology, School of Social Sciences, University of Mannheim, L13,15-17, 68131 Mannheim, Germany; 4 Department of Social Medicine, German Statutory Pension Insurance, Ruhrstr, 2, 10709 Berlin, Germany

**Keywords:** Training curriculum, Peer review, Quality assurance, Work capacity evaluation, Inter-rater reliability

## Abstract

**Background:**

The German quality assurance programme for evaluating work capacity is based on peer review that evaluates the quality of medical experts’ reports. Low reliability is thought to be due to systematic differences among peers. For this purpose, we developed a curriculum for a standardized peer-training (SPT). This study investigates, whether the SPT increases the inter-rater reliability of social medical physicians participating in a cross-institutional peer review.

**Methods:**

Forty physicians from 16 regional German Pension Insurances were subjected to SPT. The three-day training course consist of nine educational objectives recorded in a training manual. The SPT is split into a basic module providing basic information about the peer review and an advanced module for small groups of up to 12 peers training peer review using medical reports. Feasibility was tested by assessing selection, comprehensibility and subjective use of contents delivered, the trainers’ delivery and design of training materials. The effectiveness of SPT was determined by evaluating peer concordance using three anonymised medical reports assessed by each peer. Percentage agreement and Fleiss’ kappa (κ_m_) were calculated. Concordance was compared with review results from a previous unstructured, non-standardized peer-training programme (control condition) performed by 19 peers from 12 German Pension Insurances departments. The control condition focused exclusively on the application of peer review in small groups. No specifically training materials, methods and trainer instructions were used.

**Results:**

Peer-training was shown to be feasible. The level of subjective confidence in handling the peer review instrument varied between 70 and 90%. Average percentage agreement for the main outcome criterion was 60.2%, resulting in a κ_m_ of 0.39. By comparison, the average percentage concordance was 40.2% and the κ_m_ was 0.12 for the control condition.

**Conclusion:**

Concordance with the main criterion was relevant but not significant (*p* = 0.2) higher for SPT than for the control condition. Fleiss’ kappa coefficient showed that peer concordance was higher for SPT than randomly expected. Nevertheless, a score of 0.39 for the main criterion indicated only fair inter-rater reliability, considerably lower than the conventional standard of 0.7 for adequate reliability.

## Background

### Peer review

Assessing medical records in the context of a peer-review procedure is a widely used quality assurance method [1, 2]. Peer review procedures have been shown to be empirically effective, when used together with systematic feedback of results [3, 4]. The central quality criterion for uniformity of peer judgements is inter-rater reliability. Fair quality comparisons require highly independent reviews conducted by individual peers. If an individual assessment is highly reliable, there is no need for evaluation by more than one peer [5–7].

There is no generally accepted standard for interpreting inter-rater reliability; often, reliability coefficients ≥0.7 are interpreted as good [6, 8, 9]. However, reliability coefficient in peer review procedures are often lower than 0.7 [2, 7, 10–13]. Implementation of peer review offers the chance to use and process quality assurance results in internal quality management procedures [3, 14].

### Peer review in work capacity evaluation

The German Statutory Pension Insurance has developed a peer review procedure to evaluate the quality of medical experts’ reports assessing work capacity. This medical evaluation is a basic prerequisite for determining eligibility for disability benefits. Results are anchored in a medical report. The concept of peer review is based on six subsidiary quality domains and one superordinate criterion, summarized in a catalogue of 23 items. The six quality domains include significant aspects covering the quality of evaluation of work capacity, specifically: formal structure, clarity, transparency, completeness, medical-scientific principles, and the efficiency of the medical report. Each of these 22 items is reported using a four-point ordinal rating scale. The twenty-third item, the superordinate criterion, refers to the plausibility of assessment using a coherent line of reasoning (confirmability of the medical report). This item constitutes a meaningful link between individual assessment steps, from adequate diagnosis to a reasonable determination of work capacity and is rated on a three-point ordinal rating scale, represented in the three traffic light colours. If there are no interruptions in the chain of reasoning, then assessment is generally plausible (first degree of evaluation, “green”). If there are gaps in the chain of reasoning that can be filled by a physician qualified in social medicine based on the information in the report, then assessment is plausible in principle despite its limitations (second degree of evaluation, “yellow”). If there are too many gaps in reasoning that cannot be filled by a peer, then assessment is not plausible (third degree of evaluation, “red”) [15]. Peer review aims to use feedback from systematic quality evaluation to increase the transparency of reports and to generate sustainable improvements in assessments [16]. In routine application, a random sample of 140 reports from medical experts at each of the 16 regional German Pension Insurances is selected annually. The number of reports is sufficient to guarantee the required precision for quality feedback [15]. Peer review includes all written reports of personal medical examinations following an application for a disability pension. Reports are obtained from both internal and external medical experts. Reports from each regional German Pension Insurance are assessed anonymously by peers at other pension insurers based on the catalogue of 23 items. The number of participating peers per pension insurance varies and is up to the leading physician at each regional pension insurance.

During the evaluation of this item catalogue, the reliability of peer review for quality assurance was evaluated. This investigation was based on 260 medical reports from 12 participating pension insurance departments reviewed by 19 peers. Twenty reports were reviewed by all peers, while two peers reviewed 240 medical reports each. Reliability for the main target criterion (confirmability) was found to be 0.37 [9, 17]. The generally applicable standard of evaluation, 0.7, was not met. Inadequacies in peer review procedures, particularly when developing test questions and evaluation schemes (system bias), have been observed, with systematic differences among peers (individual peer bias) as a reason for low reliability. Systematic development of a review tool has been recommended to counteract system bias. To control for individual peer bias, regular training on how to use the review tool has been recommended for individuals involved in peer review [14, 18, 19].

The catalogue of peer review items was developed in a multi-level process and was pilot tested by 12 experienced physicians from 11 German Pension Insurance departments. The 12 experts were specialists in internal medicine (*n* = 3), general medicine (n = 3) and neurology/psychiatry (*n* = 3), surgery (*n* = 2) and orthopaedics (*n* = 1). A peer review manual was formulated using socio-medical expertise and was repeatedly revised. Additionally, social medical physicians involved in developing and pilot testing the peer review tool directed a two-day workshop on how discrepancies in assessing the quality criteria could be reduced, by amending the description of the criteria and/or the explanations in the manual. Discussions among peers resulted in a convergence of assessments of the superordinate criteria, indicating that the availability of a regular peer-training could reduce individual peer bias.

For this purpose, we developed a curriculum for a standardized peer-training (SPT). This study investigates, whether the SPT increases the inter-rater reliability of social medical physicians from all German Pension Insurance departments participating in a cross-institutional peer review. The success of training was evaluated by measuring inter-rater reliability (dependent variable) as a function of type of training (independent variable).

## Methods

### Developmental concept

Preliminary studies [17] and experience in peer-review procedure [20] indicate that an interactive training approach seems to be most appropriate to produce the skills required for peer review. The literature describes that learning in interactive small groups, as part of an active, collaborative, problem-based learning process, promotes an intense information processing, a deeper understanding of learning content and the possibility to check and clarify the accuracy of the understanding of learning content [21–23]. Therefore, development of training was based on the standards used when developing standardized patient education training in medical rehabilitation. This orientation towards patient training was accepted since it has been shown that patient education results in compliance, self-management and empowerment, and modifying attitudes [24]. A peer must acquire skills and competencies to perform peer review; i. e., correct application of the review criteria. These behavioural patterns are routinely required when a peer individually assesses medical experts’ reports. Furthermore, this developmental approach was chosen because published quality criteria could inform the development and implementation of the training programme [25].

Essential quality criteria include the central aspects of peer-training, as defined in a training manual, also known as a “curriculum”. The curriculum contains information on how to implement the training and defines educational objectives, target groups, teaching methodologies and requirements for the instructors. The curriculum provides guidance on how to implement the training and ensures that different instructors perform peer-training the same way [25, 26]. Based on this formal framework, an initial draft for a training programme was formulated in cooperation with a group of four social medical experts from the German Statutory Pension Insurance. These experts then also served as instructors for the SPT.

### Structure and content of the SPT

For peer-training, a curriculum consisting of two modules (basic and advanced modules) was developed and set out in a detailed training manual. The sequence of modules and their learning objectives were determined. Each module starts with an overview of basic conditions. An overview of the structure, educational objectives and methods of this training can be found in Table 1.

**Table 1:** Structure and learning objectives of peer-training

Module	Time (min)	Educational objectives	Knowledge dimensions according to Bloom’s taxonomy	Methods
basic module	90	1.1 The peer can describe^b^ the basic principles of quality assurance in work capacity evaluation	Understanding	presentation, discussion, work in small groups
	60	1.2 The peer can explain^b^ the content and structure of the peer review manual	Understanding	presentation, exercises
	30	1.3 The peer can explain^b^ the formal course of the peer review procedure in the routine application	Understanding	presentation
	45	1.4 The peer can predict^b^ the tasks in his role as a peer	Understanding	presentation, discussion
advanced module	30	2.1 The peer can apply^b^ a uniform approach in evaluating a medical report along the prescribed review items	Applying	presentation, discussion, work in small groups
	360	2.2 The peer can apply^b^ the six quality domains to individual reports by using the four-point ordinal rating scale	Applying	work in small groups
	60	2.3 The peer can apply^b^ the superordinate criterion to individual reports by using the three-point ordinal rating scale (traffic light assessment)	Applying	presentation, work in small groups
	---^a^	2.4 The peer can classify^b^ and clearly document quality deficiencies	Analysing	work in small groups
	220	2.5 The peer can independently evaluate^b^ a medical experts’ report on basis of the 23 review items	Evaluating	exercises, presentation, work in small groups

^a^Educational objective 2.4 is not associated with a specified processing time. This objective has been integrated into the educational objectives 2.2 and 2.3. While working on these two objectives, the training instructor displays adequate documentation of quality deficiencies and discusses them with the peers

^b^Key action verb associated with the knowledge dimension according to Bloom’s Taxonomy to operationalize the educational objectives

The overall three-day training course was split into nine educational objectives and included eight units lasting between 30 and 360 min. The curriculum describes the background, contents, and implementation instructions for each of these units. The curriculum also includes information on the methodical implementation of the contents of the training as well as standardized training materials. This is particularly important, as each defined educational objective is supported by an appropriate methodological and didactical approach [27, 28]. The SPT uses slides for knowledge transfer, posters for illustrations, and table templates for constructing the catalogue of 23 review items. Additionally, the SPT, uses worksheets to record problems when dealing with these items. Altogether, this results in an interactive procedure with considerable peer involvement. The following teaching modules were included:

- Brief presentation (learning objective 1.1: becoming familiar with the background of peer review)
- Discussion (learning objective 1.4: identification as a peer in a quality assurance setting)
- Work in small groups (learning objective 1.2: understanding the evaluation system)
- Exercises (learning objectives 2.1 to 2.5: use of the review items)
- Homework (learning objective 2.5: stabilisation and consistency of review outcomes)

The learning objectives were based on Bloom’s taxonomy [29]. Consecutive training included the knowledge dimensions “understanding”, to determine the meaning of peer review items; “applying”, to carry out the review procedure; “analysing”, to differentiate and categorise quality deficiencies and “evaluating”, to evaluate quality judgments independently based on medical reports [30]. Table 1 shows the assignment of learning objectives to knowledge dimensions. All learning objectives were drafted with so-called key action verbs associated with each knowledge dimensions described by Bloom’s Taxonomy. Each learning objective is further subdivided into concrete teaching contents with implementation instructions. For example, learning objective 1.2 is divided into the following subsections, including a lecture on the presentation of manual and test items of peer review for quality assurance in work capacity evaluation, a lecture on explanations of the hierarchical structure of quality criteria, a poster for visualization of the peer review, and a handout for all participants. Furthermore, a lecture presentation describes the structure of a test items including an example. An exercise worksheet describes special particularities for all 23 peer review items. This teaching content is no longer oriented towards Bloom’s Taxonomy, but rather have specific instructive character for the instructors.

Instructors were required to be individuals with broad expertise and experience in social medical assessment of the German Statutory Pension Insurance, excellent knowledge of peer review for quality assurance in evaluating work capacity and sufficient experience in managing (learning) groups. Prior to training, a one-day instructor workshop was conducted. There, the moderators discussed the learning objectives and overviewed the general training course. In addition, all medical experts’ reports processed in the SPT were evaluated by the instructors using the 23 peer review items.

The basic and advanced modules were formulated for two groups of trainees: the basic module was designed for social medicine practitioners assuming a peer role for the first time. Its main purpose is to impart knowledge about the content and structure of the peer review manual. Accordingly, learning objectives 1.1 to 1.4 are primarily presented as face-to-face lectures, supported by presentations. The advanced module was designed for social medicine practitioners who have already received training on the basic module and have acted or are acting as a peer reviewer. This module consists mostly of exercises for application of the peer review manual, using anonymised medical reports. More specifically, the evaluation system for all test questions is applied concretely and the respective assessments options are discussed.

The advanced module was designed for small groups of up to 12 peers training together using three medical reports. Each group is moderated by an experienced practitioner from the German Statutory Pension Insurance trained in social medicine. During this training, the participants practice and perform a peer review under supervision. Quality evaluations of each peer are discussed in a group to reach a group consensus when answering test questions (calibrating assessments). After training, all participating peers were sent three reports as “homework”. Using the knowledge they have gained during training, the homework involves assessing these reports based on the quality assurance manual for evaluating work capacity.

### Formative evaluation

The feasibility of the training using the manual, along with acceptance from peers, is tested as part of a formative evaluation. A total of 40 social medicine physicians from all 16 regional German Pension Insurances underwent the SPT in accordance with the peer-training curriculum. The assessment tool used for formative evaluation was based on established evaluation forms [31] and was adapted to the training curriculum. The selection, comprehensibility and subjective use of contents delivered, the trainers’ delivery and the design of training materials were assessed. A further key consideration was the subjectively estimated confidence when working with the manual. Additionally, open answer format comments on the training were gathered. The instructors (*n* = 4) evaluated integrity of treatment by assessing whether learning objectives could be established completely, partially, or not at all. A total of 36 trainees provided feedback for formative evaluation; the remaining four trainees did not return their assessment questionnaires.

### Summative evaluation

The effectiveness of the SPT was evaluated by determining the peer concordance of the superordinate criterion. Evaluation was performed using the three anonymised medical reports assessed by each peer following training. The central hypothesis was that concordance and inter-rater reliability of the superordinate criterion, the main target criterion of peer review, would be higher in the SPT than in an unstructured, non-standardized peer-training. Concordance was evaluated by measuring the percentage agreement of the peer review rating scales among peers. A two-sample t-test between proportions was performed to determine whether there was a significant difference between Intervention and control condition with respect to the cases of percentage agreement. Inter-rater reliability was calculated using Fleiss’ kappa coefficient (κ_m_) [32]. This measure of agreement considers that a certain number of opinions that agree may be coincidental and statistically random. Concordance in quality assessments was therefore tested by adjusting for chance agreement [33].

### Intervention and control condition

SPT served as the intervention group (IG), whereas unstructured, non-standardized peer-training was defined as the control group (CG). The two training conditions were implemented separately. Data from the IG were collected prospectively during this study. The IG comprised 40 social medical physicians who volunteered following an internal study announcement by the German Statutory Pension Insurance. None of these physicians was involved in developing the SPT curriculum. The participating peers had an average of 12 (SD 7.2) years of experience in the social medical service. They were specialized in surgery (*n* = 4), orthopaedics (*n* = 3), internal medicine (*n* = 20), general medicine (*n* = 3), dermatology (*n* = 1), anaesthesia (*n* = 1) and neurology/psychiatry (*n* = 8). Approximately two-thirds of these peers had long-term clinical experience in acute hospitals, rehabilitation clinics and/or outpatient care. The three-day training course was conducted according to the standardized training curriculum. During training, the peers learned basic principles of quality assurance via peer review. Only reviews from peers who had no experience with the peer review procedure and were initially trained for the first time (*n* = 26) will be included exclusively in the analysis of concordance in the IG. In order to avoid analytical bias, 14 peers who participated in the SPT were excluded, as they were already involved in the evaluation of the peer review procedure. A total of 78 reviews were included in the analysis (100% return rate).

The non-standardized peer-training (CG) took place earlier, during evaluation of the item catalogue of the peer review programme (see [17]). Nineteen social medical physicians from 12 German Pension Insurances departments were part of the CG. These peers had a similarly long experience in social medical services as the IG. They were specialized in surgery (*n* = 3), orthopaedics (*n* = 3), internal medicine (*n* = 9) and neurology/psychiatry (*n* = 4). They received a two-day training course which was one day shorter than the training for the IG. In contrast to the IG, peers were not trained in detail, nor was training constructed according to the basic structure of the peer review items. Thus, each item was provided with detailed instructions describing the rateable quality deficiencies with examples. Consequently, the non-standardized training focused exclusively on the application of the review items to small groups. In addition, no specifically prepared training materials (PowerPoint, worksheets), methods and trainer instructions were used. The reliability of the CG was determined using 56 of the 57 expected reviews (98.2%) drawn up by the peers.

The study was authorized by the German Statutory Pension Insurance based on a data protection protocol approved by the respective Department for Data Protection. Each peer participating in this project was informed of the study aims and received written information on the study procedure. All participants provided written consent before enrolment. Study participants evaluated only anonymised and declassified medical experts’ reports. Furthermore, only completely anonymised review data, with no reference to a particular medical experts’ report, were transferred to the researchers. Because this study analysed anonymous data from declassified reports no further approval was necessary.

## Results

### Feasibility and acceptance of standardized training (formative evaluation)

Peer-training was found to be feasible using the content described above during the period assessed. Although almost all educational objectives could be implemented in their entirety, educational objective 2.5 was only partially fulfilled in one working group due to lack of time. Peers reported generally high confidence in dealing with the six quality domains and the superordinate criterion while working with the review instrument (Table 2). For the superordinate criterion, i.e., assessing the confirmability of the report as a whole in accordance with quality requirements, the most frequent confidence estimation was 90% and averaged rating was 76%. The average level of confidence for handling the items of the subsidiary quality domains ranged from 70 to 84%. The participating peers regarded working with quality domain *transparency* as most uncertain; by contrast, they felt most certain when assessing the criterion *efficiency*. Overall, training implementation was regarded as successful. On a grading scale from 1 (very good) to 6 (unsatisfactory), working in small groups was particularly well regarded (average score 1.4). By contrast, the choice of medical reports for training was less well evaluated (average score 2.5). Over 90% of participating peers reported that having the chance to work on the test question catalogue in small group was fairly helpful or very helpful. Similarly, group-focused exchange of experience was rated as fairly helpful or very helpful. This point was also mentioned in the free comments section. The peers emphasized the opportunity to discuss with other peers (*n* = 11) and to work in small groups (*n* = 10). They also liked the way the groups were moderated (*n* = 5) and the inclusion of experienced social medical experts as moderators (*n* = 4). Of the participants, 94% described the training as useful and 89% would recommend it. Only two subjects mentioned that two of the three experts’ reports assessed during the SPT should be tested again for their suitability as training reports.

**Table 2:** Assessment of confidence in handling the components of the manual (*n* = 36)

Individual confidence in handling the manual regarding the…	mode	min	max	mean
…superordinate criterion: confirmability of a medical report	90	30	100	75.8
…quality domain A: formal structure	90	30	100	83.3
…quality domain B: clarity	70	40	100	73.9
…quality domain C: transparency	70	40	100	70.0
…quality domain D: completeness	80	40	100	71.7
…quality domain E: medical-scientific principles	90	20	100	79.4
…quality domain F: efficiency	90	50	100	83.9
…documentation of quality deficiencies	90	30	100	75.3

### Concordance and inter-rater reliability (summative evaluation)

The percentage agreement on the superordinate criterion of the three reports, which were evaluated by peers as “homework” following the SPT were 78.8% (report #A), 60.1% (report #B) and 41.8% (report #C), respectively, with an average of 60.2%. By comparison, the CG, which did not include curricular standardized training, reported percentage concordance of 52.9% (report #D), 35.7% (report #E) and 33.3% (report #F), with an average of 40.2% (Table 3). This difference of 20% between IG and CG was not significant at the .05 critical alpha level (t_(42)_ = 1.306, df = 42, *p* = 0.2). The inter-rater reliability of the SPT reached a κ_m_ value of 0.39, compared with a κ_m_ value of 0.12 for the CG. In addition, Fleiss’ kappa analysis showed that agreement between pairs of assessors (p_i_) was higher under both conditions than expected by chance (p_e_). The observed-chance concordance interval was three times higher in the IG than in the CG (Table 4).

**Table 3:** Percentage peer agreement of the standardized (SPT) and non-standardized (CG) peer-training

	standardized peer-training (*n* = 26)			control group (*n* = 18)		
	report #A	report #B	report #C	report #D	report #E	report #F
superordinate criterion	78.8%	60.1%	41.8%	52.9%	35.7%	33.3%
item A1	41.2%	51.9%	72.9%	53.6%	56.7%	79.5%
item A2	37.5%	46.4%	61.5%	70.6%	89.5%	79.5%
item B1	92.3%	35.0%	43.3%	79.1%	42.0%	70.8%
item B2	31.4%	25.9%	25.9%	79.1%	29.8%	30.4%
item B3	85.2%	36.2%	39.0%	100%	71.9%	35.7%
item C1	92.3%	39.6%	47.0%	30.1%	26.3%	21.6%
item C2	71.4%	62.7%	32.5%	70.6%	55.6%	70.8%
item C3	41.2%	41.9%	25.4%	69.3%	52.9%	30.4%
item D1	72.9%	28.2%	34.2%	57.5%	26.3%	31.0%
item D2	78.8%	67.5%	58.4%	78.4%	33.9%	62.0%
item D3	60.3%	72.4%	36.8%	49.7%	22.8%	23.4%
item D4	60.3%	85.8%	100%	88.2%	47.5%	77.9%
item D5	49.9%	31.3%	25.0%	45.8%	31.6%	42.7%
item E1	100%	100%	100%	88.9%	89.5%	89.5%
item E2	85.2%	32.5%	48.2%	78.4%	41.8%	42.7%
item F1	92.3%	100%	61.5%	88.9%	100%	44.4%
item F2	71.4%	25.6%	25.9%	52.9%	29.2%	70.8%

**Table 4:**
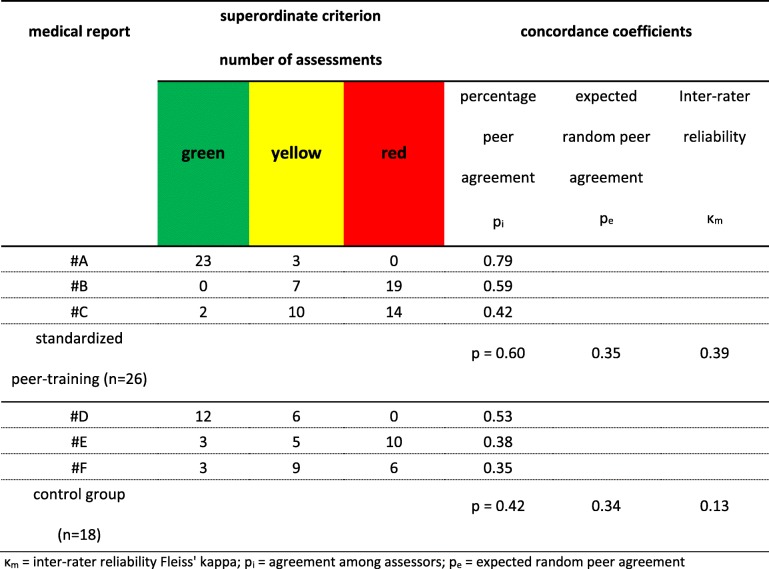
Inter-rater reliability among several assessors determined by Kappa coefficient of Fleiss (κ_m_)

## Discussion

Peer review requires well-trained peers to establish uniform standards for assessing reports as part of a cross-institutional quality assurance concept. This requires a thorough introduction to the methodologies used in the procedure. Accordingly, a curricular SPT provides knowledge not only on the review procedure to evaluate the quality of medical experts’ reports, but also on the skills used when engaging in peer activities. The review of reports assessing work capacity is a particularly challenging, as the necessary standardisation of these reports itself is limited. These reports draw on knowledge and experience from all medical fields and are therefore used to assess work capacity for specific individuals with a variety of medical conditions.

The training curriculum includes a structured manual with differentiated implementation instructions, ensuring a high level of standardisation, even across different training instructors. Having separate basic and advanced modules means the curriculum is conceptually open, allowing training on the basic module to be followed immediately or at a later time with training on the advanced module. Peer-training appears fundamental for approximation of peer judgements by social medical practitioners with experience in peer review activities. Having separate modules also means that advanced module can be offered as a stand-alone continuous professional development (CPD) course for participating healthcare professionals.

The primary activity consists of working in small group on actual reports, as adjusting peer assessments (calibration) is one of the primary aims of training. Participants were particularly positive about sharing knowledge and working jointly in groups. Furthermore, the training curriculum showed a high level of acceptance and feasibility. On average, the confidence in working with the manual was as high as 76.7%. This suggest that if levels of confidence in working with the manual were assessed immediately following training, even higher confidence might be observed, especially after working through the “homework” and regular peer activities.

Formative evaluation showed no significant needs to improve the contents of the peer-training curriculum. Care should be taken during implementation, however, to ensure that training is closely organised in accordance with the curriculum and, if necessary, to limit lengthy discussions so that all quality criteria can be discussed in detail. Accordingly, training instructors should have the soft skills required for group leadership and for implementing interactive teaching methods, and also strong expertise in social medicine and knowledge of the peer review process for quality assurance in evaluating work capacity. For implementation in routine practice two moderators per working group are recommended when teaching the advanced module, to lead the group and consensus processes and to provide substantive input from their medical expertise. Guided moderation was shown to be particularly useful when teaching the advanced module. Moderators were generally able to reconcile different review evaluations of single items among peers during discussions.

The German social security system is complex. Individuals who apply for insurance benefits due to reduced work capacity must undergo an individual personal examination lasting about 1.5 h. The medical examiner must gather all information concerning the patient’s previous job and the method by which the patient’s health problem is affected by job-related disability [34]. Although the German Statutory Pension Insurance follows common “social medicine” guidelines, the evaluation process does not have an evidence-based gold standard. As a result of the complex mix of information, different experts can come to different conclusions during the personal examination [35–39]. This can ultimately lead to disagreement between assessments of work capacity and, in some cases, to the erroneous refection of an application for disability benefits. A systematic review confirmed that physicians’ assessments of work capacity show high variability and low reliability [40]. Moderate reliability in peer review for quality assurance [17] reflects the complexity of this process. Future peer-training requires that the experts’ reports evaluated during the training course to be checked for suitability in the moderator workshop. Both good (unambiguous) reports and negative (e.g., controversial or ambiguous) examples should be used.

The superordinate criterion is defined as the main quality outcome. Meeting this criterion is considered crucial and fundamental for the validity of medical reports [17]. This criterion was therefore the main focus of this study. The six quality domains may assess relevant aspects of the quality of the reports, but not their overall usability. Although the items of the subsidiary quality domains varied widely, concordance with the superordinate criterion was nearly 20% higher under SPT (60.2%) than the control condition (40.2%). This difference is not statistically significant. According to Kirk [41] does a non-significant statistical test merely mean that we cannot exclude chance or sampling variability as an explanation for the observed differences, although a meaningful difference was found that support our initial hypothesis. Perhaps a larger sample size might have yielded to more significant results. Therefore, the best estimate of the difference of percentage agreement is 20% ± 21.3% considering a 95% confidence interval. Fleiss’ kappa coefficient also showed that peer concordance was 25% higher than the random expectation of 35%. Although medical assessments did not agree completely, concordance was descriptively higher than chance would predict, even though this difference was not significantly higher due to the small number of reports. For all three reports, the concordance of the superordinate criterion was higher in the IG than in the CG (see Table 3). The superordinate criterion during SPT showed only fair inter-rater reliability (κ_m_ = 0.39) and was substantially lower than the conventional standard of 0.7 for adequate reliability. However, other studies showed similar or even lower inter-rater reliability [11, 13, 18, 42–46]. A meta-analysis reported an average weighted Kappa of 0.31 [47], whereas a systematic review found inter-rater agreements in evaluation of disability that ranged from 0.10 to 0.86 [40]. During the initial evaluation of the catalogue of peer review items, reliability values of 0.37 were calculated [17], indicating that a single training session cannot immediately increase inter-rater reliability.

### Limitations

This study provided evidence of the effectiveness of peer-training in a realistic setting rather than proving efficacy. The CG differed from the IG in that they received non-standardized peer-training and evaluated different anonymized medical reports. To ensure comparability, both groups would need to use the same reports. For organisational reasons, however, all participants were trained using the standardized training course. Immediately after evaluation, routine implementation of the peer review process was started. Retraining of a potential CG was not possible. Therefore, the IG that was trained at a different time without a standardized training manual uses different reports. Differences in reviewed reports can lead to a different correspondence among peers. Because each report involves an individual case history, medical reports cannot be directly compared with each other. Experts determine work capacity on a case-by-case basis. Accordingly, inter-rater reliability of the training courses can only be compared and interpreted indirectly, because variability may be due to the reports themselves. A bias due to the use of different medical reports in the two training sessions (IG and CG) cannot be finally ruled out. Further, the practical constraints of the peer review programme for quality assurance did not allow for allocation of individual reviewers or for anonymised medical reports chosen at random. The developed training does not include tests on learning objectives. Rather, training is based on the common application of quality criteria for reports under the supervision of an instructor. Assessments of learning objectives after basic training may improve the ability to use and interpret the review items. The advanced training module is based on the evaluated medical reports. The learning objectives of this module are based exclusively on the practical application of the 23 review items in small groups and cannot be tested in a standardized way. Further operationalisation of the learning objectives and the subordinate teaching content is reasonable for the future development of SPT. This would allow success monitoring at several points in the training, which might be associated with an improvement of peer agreement.

A further fundamental limitation of the training success, however, was the reviewed reports themselves. In Germany, work capacity is directly assessed by medical experts. However, there may be discrepancies between the formal definition of work ability being evaluated and the actual criteria of the evaluating experts. The formal criterion for work ability in Germany is based on the number of hours per day a person could work (> 6 h, 3–6 h or < 3 h). In practice, this strict classification cannot always be quantified on the basis of functional limitations. Accordingly, it is possible that during personal examination, the medical expert decide on work capacity according the individual case and to a rule of thumb [34, 48]. If the assessment of work capacity is not objectively comprehensible, peers can only make difficult uniform assessments during peer review or peer-training. This may also result in low post-training reliability.

## Conclusion

Peer-training prepares participating social medical practitioners for their role as peers and creates common evaluation standards. Because the German Statutory Pension Insurance desires nationwide cross-institutional application of peer reviews, the advanced module should be offered regularly (e.g. annually) as a stand-alone CPD measure for healthcare professionals active in quality assurance procedures. Annual review will maintain the effects of training and minimise peer bias. As peers usually remain the same, their experience will increase with each training session and peer review. This will have positive effects on inter-rater reliability. Evidence of this assumed effect is only verifiable with continuous re-evaluation. If reliability does not improve after repeated training, the review instrument itself should be redesigned. Following evaluation of the peer review manual [17], the German Statutory Pension Insurance considered the development of a standardized peer curriculum as a further component in implementing a quality assurance system in evaluating work capacity based on social medical experts’ reports.

## Abbreviations

CG: Control group
CPD: Continuous professional development
IG: Intervention group
max: Maximum
min: Minimum
p_e_: Expected random peer agreement
p_i_: Agreement among assessors
SD: Standard deviation
SPT: Standardized peer-training
κ_m_: Fleiss’ kappa

## References

[1] Shaw C (2001). External assessment of health care. BMJ.

[2] Lilford R, Edwards A, Girling A, Hofer T, Di Tanna GL, Petty J (2007). Inter-rater reliability of case-note audit: a systematic review. J Health Serv Res Policy.

[3] Edwards MT (2011). The objective impact of clinical peer review on hospital quality and safety. Am J Med Qual.

[4] Glattacker M, Jäckel W (2007). Evaluation of quality assurance - current data and consequences for research. Gesundheitswesen.

[5] Wirtz M, Kutschmann M (2007). Analyzing interrater agreement for categorical data using Cohen’s kappa and alternative coefficients. Rehabilitation.

[6] Wirtz M, Casper F (2002). Beurteilerübereinstimmung und Beurteilerreliabilität.

[7] Goldman RL (1992). The reliability of peer assessments of quality of care. JAMA.

[8] Altman DG. Practical statistics for medical research: Chapman and Hall; 1991.

[9] Landis JR, Koch GG (1977). The measurement of observer agreement for categorical data. Biometrics.

[10] Hofer TP, Asch SM, Hayward RA, Rubenstein LV, Hogan MM, Adams J (2004). Profiling quality of care: is there a role for peer review?. BMC Health Serv Res.

[11] Neuderth S (2004). Externe Qualitätssicherung durch Peer-Review.

[12] Farin E, Carl C, Lichtenberg S, Jäckel WH, Maier-Riehle B, Rütten-Köppel E (2003). Evaluating the rehabilitation process by means of peer review: examination of the methods used and findings of the 2000/2001 data collection in the somatic indications. Rehabilitation.

[13] Kadar N (2010). Systemic bias in peer review: suggested causes, potential remedies. J Laparoendosc Adv Surg Tech A.

[14] Smith MA, Atherly AJ, Kane RL, Pacala JT (1997). Peer review of the quality of care. Reliability and sources of variability for outcome and process assessments. JAMA.

[15] Deutsche Rentenversicherung Bund. Qualitätssicherung der sozialmedizinischen Begutachtung: Manual zum Peer Review-Verfahren. Berlin: Deutsche Rentenversicherung Bund; 2013. Available from: http://www.deutsche-rentenversicherung.de/Allgemein/de/Inhalt/3_Infos_fuer_Experten/01_sozialmedizin_forschung/downloads/sozmed/begutachtung/manual_peer_review.pdf?__blob=publicationFile&v=5.

[16] Legner R, Cibis W (2007). Quality Assurance in Sociomedical Evaluation. Rehabilitation.

[17] Strahl A, Gerlich C, Wolf H-D, Gehrke J, Müller-Garnn A, Vogel H (2016). Quality Assurance in Sociomedical Evaluation by peer review: a pilot project of the German statutory pension insurance. Gesundheitswesen..

[18] Harris CD, Bratzler DW (2013). Evaluating quality of care: the role of peer review. J Okla State Med Assoc.

[19] Tuijn S, Janssens F, Robben P, van den Bergh H (2012). Reducing interrater variability and improving health care: a meta-analytical review. J Eval Clin Pract.

[20] Klosterhuis H (2010). Rehabilitation quality assurance of the German pension insurance - a critical review. RVaktuell.

[21] Jones RW (2007). Learning and teaching in small groups: characteristics, benefits. problems and approaches Anaesth Intensive Care.

[22] Springer L, Stanne ME, Donovan SS (1999). Effects of small-group learning on undergraduates in science, mathematics, engineering, and technology: a meta-analysis. Rev Educ Res.

[23] Prince M (2004). Does active learning work? A Review of the Research. J Eng Educ.

[24] Faller H, Reusch A, Ströbl V, Vogel H (2008). Patient education as a constituent of a patient-oriented approach in rehabilitation. Rehabilitation.

[25] Ströbl V, Küffner R, Müller J, Reusch A, Vogel H, Faller H (2009). Patient education: quality criteria in ist implementation. Rehabilitation.

[26] Ströbl V, Friedl-Huber A, Küffner R (2007). Beschreibungs- und Bewertungskriterien für Patientenschulungen. Prax Klin Verhal und Rehabil.

[27] Möller C (1973). Technik der Lernplanung: Methoden und Probleme der Lernzielerstellung.

[28] Faller H, Reusch A, Meng K (2011). DGRW-update: patient education. Rehabilitation.

[29] Bloom BS, Engelhart MD, Furst EJ, Hill WH, Krathwohl DR (1956). Taxonomy of educational objectives: The classification of educational goals. Handbook 1: Cognitive domain.

[30] Krathwohl DR (2002). A revision of Bloom’s taxonomy: an overview. Theory Pract.

[31] Meng K, Seekatz B, Roßband H, Worringen U, Faller H, Vogel H (2009). Development of a standardized back School for in-Patient Orthopaedic Rehabilitation. Rehabilitation.

[32] Fleiss J (1971). Measuring nominal scale agreement among many raters. Psychol Bull.

[33] Bortz J, Lienert GA. Kurzgefasste Statistik für die klinische Forschung: Leitfaden für die verteilungsfreie Analyse kleiner Stichproben; mit 97 Tabellen sowie zahlreichen Formeln. Springer; 2008.

[34] Aurich-Beerheide P, Brussig M (2017). Assessment of work ability in competing strands of social insurance: the German case. J Poverty Soc Justice.

[35] Tait RC, Chibnall JT, Miller L, Werner CA (2011). Judging pain and disability: effects of pain severity and physician specialty. J Behav Med.

[36] Chibnall JT, Dabney A, Tait RC, Cedraschi C, Nordin M, Nachemson A (2000). Internist judgments of chronic low back pain. Pain Med.

[37] Tait RC, Chibnall JT (1997). Physician judgments of chronic pain patients. Soc Sci Med.

[38] Merten T, Friedel E, Mehren G, Stevens A (2007). Negative response bias and the validity of personality profiles in neuropsychiatric assessment. Nervenarzt.

[39] Stevens A, Friedel E, Mehren G, Merten T (2008). Malingering and uncooperativeness in psychiatric and psychological assessment: prevalence and effects in a German sample of claimants. Psychiatry Res.

[40] Barth J, de Boer WEL, Busse JW, Hoving JL, Kedzia S, Couban R (2017). Inter-rater agreement in evaluation of disability: systematic review of reproducibility studies. BMJ.

[41] Kirk RE (1996). Practical significance: a concept whose time has come. Educ Psychol Meas.

[42] Abragam A, Lincke H-O, Lux A, Wallesch CW (2002). Peer review of routine clinical case reports - an instrument of quality management? Results of a pilot investigation. Nervenarzt.

[43] Dharmar M, Marcin JP, Kuppermann N, Andrada ER, Cole S, Harvey DJ (2007). A new implicit review instrument for measuring quality of care delivered to pediatric patients in the emergency department. BMC Emerg Med.

[44] Hayward RA, Bernard AM, Rosevear JS, Anderson JE, McMahon LF (1993). An evaluation of generic screens for poor quality of hospital care on a general medicine service. Med Care.

[45] Kameoka J, Okubo T, Koguma E, Takahashi F, Ishii S, Kanatsuka H (2014). Development of a peer review system using patient records for outcome evaluation of medical education: reliability analysis. Tohoku J Exp Med.

[46] Smith MA, Atherly AJ, Kane RL, Pacala JT, Dans PEWJOS, RL G (1997). Peer review of the quality of care. JAMA.

[47] Goldman RL (1994). The reliability of peer assessments. A meta-analysis. Eval Health Prof.

[48] Geiger BB, Garthwaite K, Warren J, Bambra C. Assessing work disability for social security benefits: international models for the direct assessment of work capacity. Disabil Rehabil. 2017. 10.1080/09638288.2017.1366556. 10.1080/09638288.2017.1366556 28841811

